# Epigenetic Heterogeneity in Human Colorectal Tumors Reveals Preferential Conservation And Evidence of Immune Surveillance

**DOI:** 10.1038/s41598-018-35621-y

**Published:** 2018-11-23

**Authors:** Marc D. Ryser, Ming Yu, William Grady, Kimberly Siegmund, Darryl Shibata

**Affiliations:** 1 Department of Surgery, Division of Advanced Oncologic and GI Surgery, Duke University Medical Center, Durham, NC United States; 2 Department of Mathematics, Duke University, Durham, NC United States; 3 Department of Population Health Sciences, Duke University Medical Center, Durham, NC United States; 4 Clinical Research Division, Fred Hutchinson Cancer Research Center, Seattle, WA United States; 5 Department of Medicine, University of Washington School of Medicine, Seattle, WA United States; 6 Department of Preventive Medicine, University of Southern California Keck School of Medicine, Los Angeles, CA United States; 7 Department of Pathology, University of Southern California Keck School of Medicine, Los Angeles, CA United States

## Abstract

Genomic intratumoral heterogeneity (ITH) is common in cancers, but the extent of phenotypic ITH is uncertain because most subclonal mutations are passengers. Since tumor phenotypes are largely driven by epigenetics, methylomic analyses can provide insights into phenotypic ITH. Following this principle, we determined the extent of epigenetic ITH in 16 human colorectal tumors by comparing the methylomes from spatially separated regions in each tumor. Methylomes from opposite tumor sides were similar (Pearson correlation >0.95) with little evidence of ITH or stepwise selection during growth, suggesting that the epigenome of a sampled tumor largely reflects that of its founder cell. Epigenetic conservation was functional, with higher conservation at promoters and expressed genes compared to non-coding regions. Despite epigenomic conservation, RNA expression varied between individual tumor glands, indicating continued adaption during growth. Because many promoters and enhancers were unmethylated, continued adaptation may be due to phenotypic plasticity. Gene enrichment analyses identified that interferon signaling and antigen-processing and presenting pathways were strongly conserved during tumor growth, suggesting a mechanism for immune evasion. In summary, our findings suggest that epigenomes are preferentially conserved during tumor growth and that early tumor cells are poised for rapid growth, phenotypic adaptation, and immune evasion.

## Introduction

Multiregional sampling reveals that genomic intratumor heterogeneity (ITH) is common in many human tumors^1–3^, yet its clinical significance remains uncertain^4^. A possible interpretation of genomic ITH is that of ongoing clonal evolution, where successively more fit subclones with different mutations replace less fit subclones. However, most subclonal mutations are passengers^5^ and it is unclear how or if subclonal mutations confer selection during growth. For instance, quantitative analyses of human colorectal tumors revealed topographic patterns of genetic alterations that are consistent with single expansions of neutral evolution rather than stepwise selection during growth^6–8^. More generally, mutation allelic frequencies of several solid tumor types have been shown to be consistent with single neutral expansions during growth^9^.

**Figure 1:**
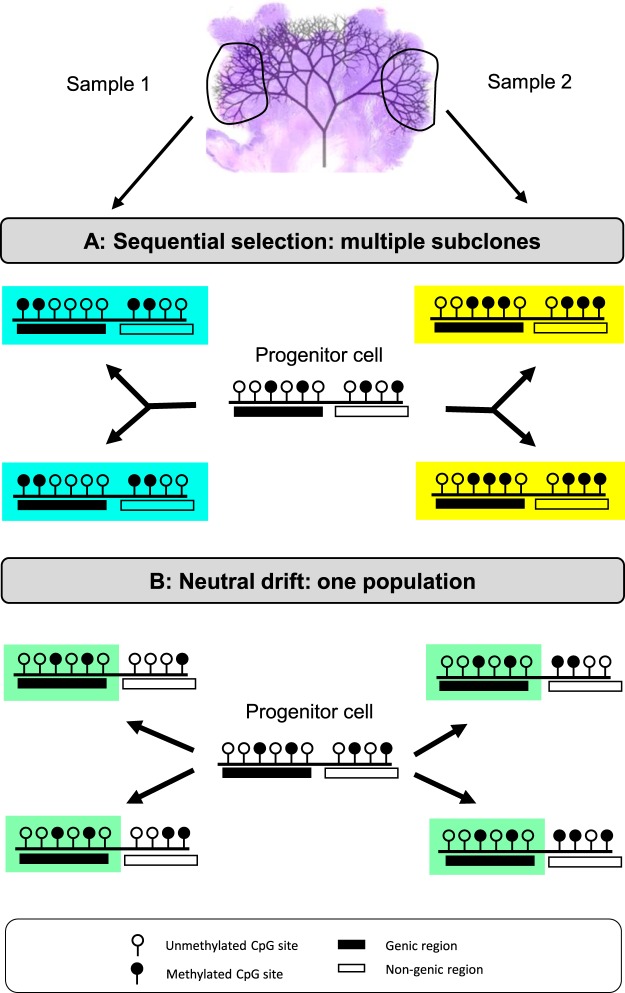
Stepwise selection vs single expansion. Sampling opposite tumor sides provides ancestral information on how human tumors grow. We distinguish two types of measurable epigenetic ITH: spatial heterogeneity between separated tumor regions, and preferential conservation along the genome. Sequential stepwise selection and single expansion dynamics have different implications for spatial ITH and preferential conservation. (**A**) With sequential stepwise selection during growth, spatial ITH is sectored, with neighboring cells more related than distant cells. Preferential conservation along the genome is not observed because sweeps eliminate differential drift due to epigenetic replication errors. (**B**) With single expansions and neutral drift, spatial ITH is expected to be low across the entire tumor. Preferential conservation along the genome arises because epigenetic replication errors accumulate at non-functional CpG sites. Filled circles represent methylated CpG sites.

To date, little is known to what extent genomic ITH is translated into phenotypic heterogeneity. Epigenomic variations can lead to phenotypic ITH even in absence of genetic driver mutations, and phenotypic ITH may in turn drive clinical progression of the tumor. In practice, multiregional epigenomic comparisons can be used to infer the degree of phenotypic heterogeneity because DNA methylation of enhancers, promoters, and gene bodies modulates gene expression^10–12^. Epigenomes should differ between tumor sides if distinct subclones with fitter phenotypes emerge stepwise during growth (Fig. 1A). In contrast, with single neutral expansions, epigenomes should be similar between sides because the tumor is essentially a single population of cells with similar phenotypes (Fig. 1B).

To evaluate the degree of phenotypic ITH in human colorectal tumors, we sampled and compared the epigenomes from opposite sides of 16 specimens (Fig. 1). Epigenetic ITH was found to be low, with little evidence of recent stepwise selection. In addition, epigenetic conservation was nonuniform along the genome, with preferential conservation in genes that are functionally important.

## Results

### Epigenomes are similar between opposite tumor sides

**Figure 2:**
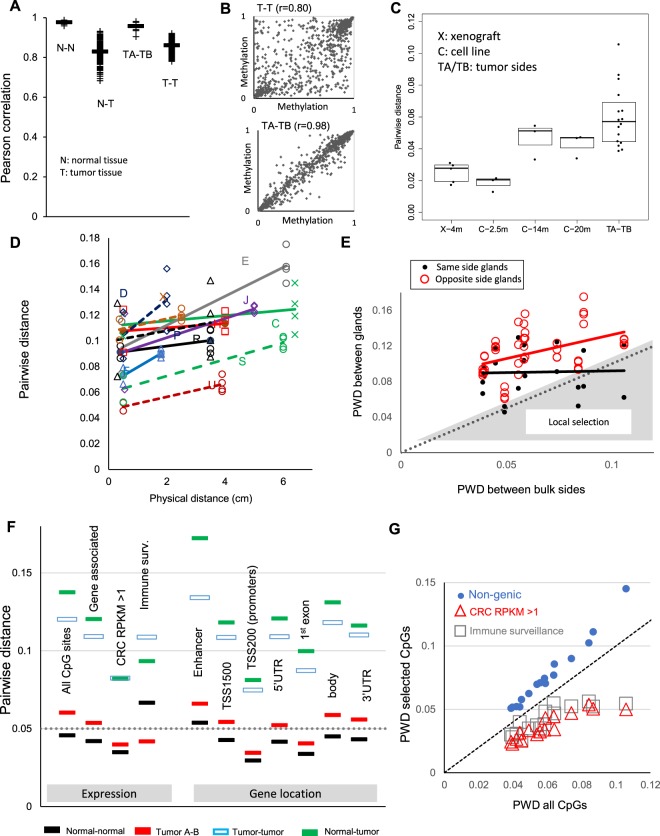
Minimal spatial ITH with preferential conservation along the genome. (**A**) Pairwise similarity of methylomes as measured by Pearson correlation. Methylomes are more alike between spatially separated samples of the same tumor (TA-TB) and between normal colons of different patients (N-N) than between tumor and normal of the same patient (N-T) and between tumors of different patients (T-T). Correlations varied across the four groups (Kruskal-Wallis test, p < 0.0001). All between-group comparisons were strongly significant (Wilcoxon rank sum test, p < 0.0001) except for N-N vs TA-TB (p = 0.004) and N-T vs T-T (p = 0.02). (**B**) Comparison of methylation levels between tumor sides (TA-TB) and between different tumors for a subset of CpG sites in tumor R, r: Pearson correlation. (**C**) Pairwise distances (PWD) between DNA methylation of xenografts (X) after ~4 months (m) originating from a single cell were small. PWDs between parallel serial tissue culture cell lines originating from a single cell (**C**) were small at 2.5 months but significantly increased after 14 months (Wilcoxon rank sum test, p = 0.02). PWDs between opposite tumor sides in human tumors (TA-TB) were greater than the PWDs in the combined xenograft and cell line experiments (p = 0.04). (**D**) Methylation differences between glands on the same tumor side (<0.5 cm apart) and between glands on opposite sides (>1.8 cm apart) were similar. PWDs between glands within a tumor side were all greater than the four-month-old xenografts. Letters designate tumors. (**E**) PWDs between glands on opposite sides of the same tumor were generally greater than the PWDs of their bulks (red circles). Comparing the PWDs of glands on the same tumor side against the PWDs of the tumor bulks (black dots) revealed 4 of the 22 gland pairs with lower PWDs, possibly indicating a localized selective sweep (shaded triangle). (**F**) Mean PWDs between normal colons of different patients (black), between spatially separated tumor samples in the same patient (red), tumor samples from different patients (blue-white), and between normal and tumor samples from the same patient (green). Greater conservation between tumor sides (red) is seen for highly expressed genes in colorectal cancer (CRC RPKM > 1) and at promoters (TSS200). Conservation was generally greater between normal colons than between tumor sides. An exception was a subset of immune surveillance antigen processing genes that were more conserved between tumor sides than between normal colons (Wilcoxon rank sum test, p < 0.0001). The full PWD distributions are shown in Fig. S2. (**G**) For each tumor (n = 16), PWDs of select CpG sites are compared against the PWDs of all CpG sites. Drift is greater at non-genic (passenger) CpG sites but preferential conservation is seen at CpG sites associated with highly expressed genes in colorectal cancer (CRC RPKM > 1) and a subset of IS genes associated with antigen presentation.

DNA methylation was measured at ~850,000 CpG sites from bulk samples of six normal colons and opposite sides of 16 human colorectal tumors (Table 1; 4 adenomas and 12 colorectal cancers (CRCs)). As seen in other studies^13–15^, there were substantial differences between the epigenomes of tumor and normal tissue within individual patients, and between tumors of different patients (Fig. 2A,B). In contrast, the epigenomes between normal colons of different patients and between opposite sides of the same tumors were strongly conserved (Pearson > 0.95). These findings suggest that epigenomes evolve during progression from normal colon to tumor, but are more conserved between two sides of the same tumor.

**Table 1:** Colorectal tumors.

Tumor	Type (stage)	Size (cm)	Matched normal
K	Adenoma	6.0	No
S	Adenoma	6.0	No
P	Adenoma	3.5	No
X	Adenoma	2.5	No
O	Cancer(3)	9.5	No
C	Cancer(3)	6.4	Yes
H	Cancer(4)	4.0	Yes
J	Cancer(3)	5.0	Yes
M	Cancer(2)	3.0	No
T	Cancer(3)	5.7	No
W*	Cancer(1)	3.4	Yes
U	Cancer(2)	3.9	No
D	Cancer(1)	2.0	No
F	Cancer(1)	1.8	No
E**	Cancer(1)	6.1	Yes
R	Cancer(1)	3.5	Yes

*MSI+; **POLE mutant.

### Colorectal tumors consist of older cell populations

Other studies have documented that epigenetic ITH is common in CRCs^14–16^, but the biological significance is uncertain, especially because even small epigenetic changes may cause phenotypic differences. Here we sought to determine whether epigenomes and their phenotypes were sufficiently different to confer selection. Epigenomes can detect recent selection because, like DNA sequences, epigenetic patterns are copied almost exactly between generations (Fig. 1A). Cells of a recent expansion initially have similar epigenomes that they inherit from their founder cell, but they become polymorphic with time (“molecular clock hypothesis”^17^). DNA methylation replication error rates are up to 10,000 times higher than DNA mutation rates^17^ and therefore can record very recent selective sweeps.

To benchmark the tempo of epigenetic changes, epigenomes were compared between different parts of experimental tumors – both xenografts and *in vitro* cell lines – started from single cells. Although these experimental “clonal” tumors do not fully model sporadic tumorigenesis and lack immune systems, they can calibrate how fast epigenomes diversify after the start of a clonal expansion. Clonal xenografts grown on different mouse flanks had minimal pairwise differences (PWD) after 4 months (Fig. 2C). Consistent with a molecular clock, clonal serial parallel cell lines originating from a single cell had low PWDs at 2.5 months, and substantially higher PWDs after 14 and 20 months. Compared to these experimental tumors, PWDs between tumor sides were greater than between single cell xenografts but overlapped with the PWDs between older single cell serial parallel cultures. Therefore, the colorectal tumors appear to be older than 4 months of age by the time of surgery and there is no evidence of very recent selective sweeps across the entire tumor specimens. Indeed, such sweeps would have led to lower PWDs between opposite tumor sides compared to the PWDs in experimental systems.

### Multiregional gland sampling reveals little evidence of recent selection

To search for smaller localized subclonal sweeps within tumor regions, we sequenced the methylomes of gland pairs from opposite sides of 11 tumors (four glands per tumor). Neighboring glands within bulk regions (<0.5 cm apart) all had PWDs (Fig. 2D) greater than the four-month old xenografts (Fig. 2C) and were almost as related as glands centimeters apart on opposite tumor sides (Fig. 2D). This lack of correlation between physical and epigenomic distances is more consistent with single expansions (Fig. 1B) rather than active ongoing selection, where neighboring glands should be much more related than distant glands (Fig. 1A). PWDs between glands were generally greater than between bulk tumor sides (Fig. 2E), likely reflecting that population averages drift slower than smaller tumor gland subpopulations. Four of the 22 gland pairs within a tumor side had PWDs smaller than between their bulk tumor sides, which could reflect regional subclonal selective sweeps during growth. Any such selection did not appear recent because PWDs between glands were all greater than the four-month old xenografts (Fig. 2C).

### Preferential epigenetic conservation at functional regions

The relatively uniform spatial epigenetic ITH within and between tumor regions indicates drift rather than stepwise selection is the major driver of epigenetic ITH. For a single neutral expansion, the epigenome of the tumor should resemble the epigenome of its founder cell. Furthermore, subsequent drift during growth is expected to be nonuniform along the genome, with preferential conservation at functional regions because DNA methylation modulates expression^10,12^ (Fig. 1B). More precisely, methylation changes in non-genic regions are expected to be neutral (passengers) whereas methylation changes in expressed genes are expected to be subject to positive or negative selection. Consistent with this drift hypothesis, we found increased conservation at gene-associated CpG sites, with preferential conservation at more highly expressed genes and a subset of genes associated with immune surveillance (Fig. 2E). Within genes, there was greater conservation at promoters (TSS200) and in first exons. Conserved methylation therefore identifies regions in a founder cell epigenome that are selectively maintained during growth, in contrast to non-functional regions that are subject to continued drift (Fig. 2F).

### A core set of conserved genes

**Figure 3:**
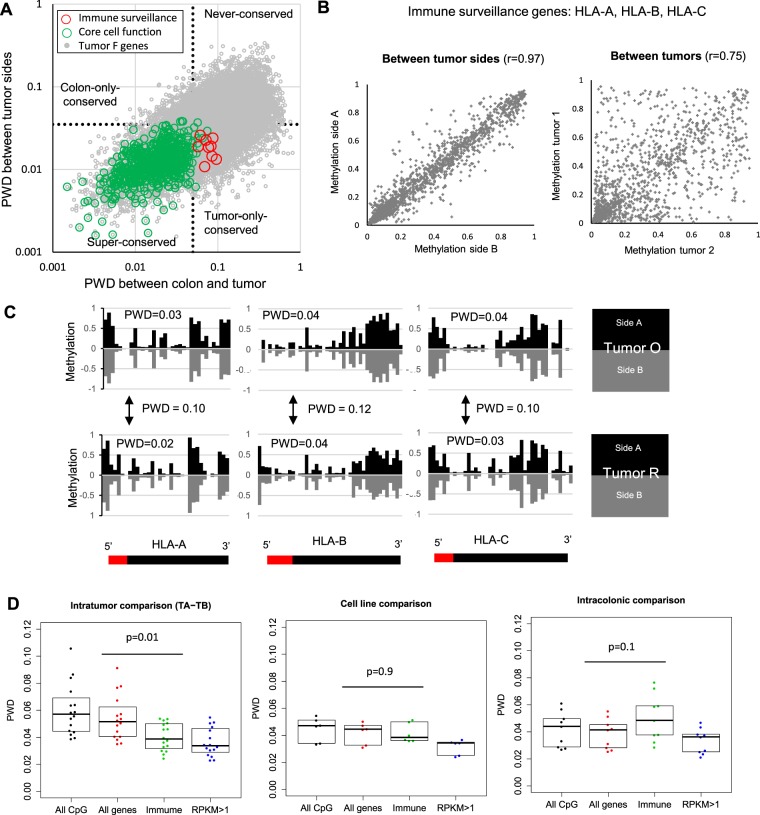
Preferential epigenetic conservation. (**A**) Super-conserved genes have similar methylation between tumors and normal. These genes are enriched in core cell functions (green circles, Table 2). Tumor-only-conserved genes are enriched for immune surveillance (IS) genes (red circles). Never-conserved genes tend to be non-expressed. Tumor F data (all genes in grey) are illustrated. (**B**) Example of greater CpG site methylation conservation for three IS genes (HLA-A, HLA-B, HLA-C) between tumor sides compared to between tumors. r: Pearson correlation. (**C**) Methylation patterns (5′ to 3′, red bar indicates the promoter) at HLA-A, HLA-B, and HLA-C are preferentially conserved between sides of the same tumor but are more different between individual tumors (shown for tumors O and R). (**D**) IS gene methylation conservation between tumor sides (TA-TB) is greater compared to all genes (Wilcoxon rank sum test, p = 0.01). This preferential conservation is not seen in the parallel cell lines at 14 and 20 months (p = 0.93) or between four different parts of 9 adult normal colons where IS genes methylation was less conserved compared to all genes (p = 0.14).

Preferential epigenetic conservation enabled us to identify genes that were under greater selection during growth. Among the 22,430 annotated genes with four or more CpG sites, we identified four subgroups based on pairwise distances between tumor sides and between normal colons of different patients: super-conserved genes (conserved between tumor sides and between normal colons), tumor-only conserved, normal colon-only conserved and never-conserved (Fig. 3A, Table 2). Super-conserved genes (n = 2,769) were defined as having very similar epigenomes between tumor sides (PWD < 0.05) in 90% or more of tumors, and between normal colons of different patients (PWD < 0.075), which corresponds to little change during progression and growth. Among super-conserved genes, a pathway enrichment analysis with Reactome^18^ identified significant enrichment (FDR < 0.05) for 612 loci involved in 66 pathways important for general cell metabolism. Many genes involved with core cell functions appear to be under selection in normal colon and tumors because their methylation remains conserved during progression (Table 2).

**Table 2:** Conserved genes.

Conservation	Loci (n)	Pathway enriched loci	Enriched Reactome Pathways
Super-conserved* TA-TB: PWD < 0.05 N-N: PWD < 0.075	2,769	612 (66 pathways)	Cell cycle DNA replication and repair RNA and protein metabolism Chromatin organization Gene expression
Normal only* TA-TB: PWD > 0.05 N-N: PWD < 0.075	0		
Never conserved* TA-TB: PWD > 0.05 N-N: PWD > 0.075	2,977	32 (1 pathway)	Collagen chain trimerization
Tumor only* TA-TB: PWD < 0.05 N-N: PWD > 0.075	242	9 (9 pathways) HLA-A PSMD5 FLNB FZR1 RNF114 TRIM4 CUL3 ARIH2 UBE2O	Immune system: - Endosomal/Vacuolar pathway - Antigen Presentation: Folding, assembly and peptide loading of class I MHC - Interferon alpha/beta signaling - ER-Phagosome pathway - Antigen processing-cross presentation - Interferon gamma signaling - Interferon Signaling - Class I MHC antigen processing & presentation - Immunoregulatory interactions between a lymphoid and a non - lymphoid cell
Tumor only, extended** TA-TB: PWD < 0.05 T-T: PWD > 0.075 N-T: PWD > 0.075 N-N: PWD > 0.075	1,767	19 (10 pathways) B2M CYBA FLNB GBP4 HLA-A HLA-B HLA-C HLA-F HLA-H IRF7 ISG20 ITGB5 KPNA1 NAT1 OAS1 PSMD5 SOCS3 TRIM2 TRIM6	Immune system: - Antigen Presentation: MHC I - Endosomal/Vacuolar pathway - Interferon alpha/beta signaling - Interferon gamma signaling - Antigen processing-Cross presentation - ER-Phagosome pathway - Interferon Signaling - MHC I antigen processing, presentation - Interactions Lymph & non-Lymph cell - Cytokine Signaling Immune system

*The PWD criteria were required to be satisfied for ≥90% of comparisons; **Average PWDs were used for inclusion criteria.

TA: tumor side A; TB: tumor side B; N: normal colon; T: tumor.

No loci were consistently conserved only between normal colons but not between tumor sides, indicating that most genes that are highly regulated in the colon are also under selection during growth. A total of 2,977 loci were conserved neither between tumor sides nor between normal colons (PWD > 0.05 between tumor sides in >90% of tumors, PWD > 0.075 between normal colons), with significant nonsensical enrichment in a collagen chain trimerization pathway.

### Preferential tumor-specific conservation of immune surveillance genes

Areas of the epigenome that are only conserved between tumor sides and not between normal colons can potentially identify which genes and pathways are uniquely important for tumorigenesis. There were 242 loci consistently conserved only in tumors (PWD < 0.05 between tumor sides in >90% of tumors) but not between normal colons (PWD > 0.075), and pathway analyses with Reactome revealed significant enrichment of 9 genes and 9 immune surveillance (IS) pathways (Table 2).

To facilitate conservation analyses of individual tumors, we introduced an extended tumor-only classification that filtered out genes that are inherently epigenetically stable. More precisely, across all tumors, we identified 1,767 genes that were conserved between tumor sides (average PWD across all tumors <0.05) but not conserved between normal colon and tumor of the same patient (average PWD > 0.075), normal colons of different patients (average PWD > 0.075), and tumors of different patients (average PWD > 0.075). In this extended tumor-only group, there was again significant pathway enrichment of 19 tumor-specific IS genes involved in antigen-presentation and processing, and in interferon signaling (Table 2). Next, among the 1,767 genes in the extended tumor-only group, we focused on individual tumors and identified which genes were conserved between the sides of individual tumors (tumor-specific PWD < 0.05). Through this process, IS gene enrichment was found in 15 of the 16 tumors (Table S1). The only tumor without preferential IS gene conservation enrichment had a POLE mutation, suggesting an alternative mechanism for immune evasion when the mutation burden is exceptionally high.

The preferential conservation of IS genes between different regions of the same tumor is illustrated on the examples of HLA-A, HLA-B, and HLA-C in Fig. 3C,D. IS epigenetic conservation within tumors is greater than between tumors or between multiple samples of individual colons (Fig. 3E). Tumor-specific preferential conservation of antigen processing and presentation genes suggests immune surveillance exerts strong selective pressures on most human colorectal tumors during growth.

Tumor-specific conservation is likely due to selection, but other factors such as greater replication fidelity could also explain a lack of drift. One way to test for selection is to determine whether IS gene conservation persists in cell lines which are not under immune surveillance. Unlike between tumor sides, IS genes were not preferential conserved in the clonal parallel cell lines relative to all genes (Fig. 3E), indicating that methylation at these genes drift more in the absence of an immune system.

### Immune surveillance gene methylation differs within healthy adult colons

Patient-specific somatic mutations in human tumors lead to the expression of patient-specific neoantigens, which in turn are presented to the immune system. This can lead to immunoediting, whereby the immune system removes certain neoantigen-presenting cancer cells^19^. The conservation of IS genes as observed in our study samples may prevent immunoediting and enable the tumor to grow despite active immune surveillance. More precisely, tumor-specific IS gene conservation may indicate that highly specific processing and presentation of neoantigens may enable tumors to avoid immunoediting. To test this hypothesis, we performed multiregional measurements of methylation in healthy colons, where crypts independently accumulate many different mutations with aging^20^. If IS gene methylation customizes the presentation of specific neoantigens, then conservation should not be observed between different colonic regions because they have acquired different somatic mutations and hence neoantigens over time. Consistent with this hypothesis, while the methylation of highly expressed genes was conserved, IS gene methylation drifted between the four different parts each of nine healthy adult colons (Fig. 3E). In summary, the difference in IS gene conservation in tumors compared to healthy colons (Fig. 3E) suggests that each tumor may be locked into its own IS gene methylation pattern that enables successful evasion of immunoediting.

### Phenotypic plasticity despite epigenetic conservation

In most human tumors, many different microscopic phenotypes can be observed. In the selective sweep scenario (Fig. 1A), different phenotypes can arise by stepwise selection. In the single expansion scenario (Fig. 1B), phenotypic heterogeneity can arise thanks to phenotypic plasticity^21,22^ which allows cells with similar epigenomes to express multiple phenotypes. Such phenotypic plasticity is observed in the normal colon where microscopically different cell types in a single crypt have nearly identical epigenomes^23,24^. In particular, broadly permissive^24^ or open chromatin at enhancers and promoters license plasticity because transcription factors induced by the microenvironment determine final cell phenotypes.

**Figure 4:**
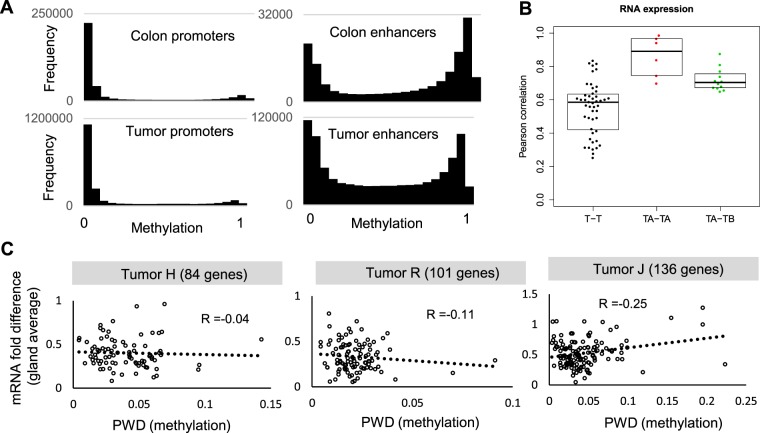
Phenotypic plasticity. (**A**) Most promoters were demethylated in both normal colons and tumors, indicating open configurations. Enhancer methylation was bimodal in normal colon and more variable in tumors, with proportionally fewer tumor enhancers in fully methylated or closed configurations. Analyses included all enhancer or promoter CpG sites for the six normal colons and 16 tumors (both sides). (**B**) RNA expression levels were more similar between glands in the same tumor side (TA-TA) compared to glands on opposite tumor sides (TA-TB) (Wilcoxon rank sum test, p = 0.013) or between glands of different tumors (T-T) (p < 0.0001). Analyses based on 4 glands from 3 tumors. (**C**) No correlation between gene epigenetic conservation and expression level variability.

In our study samples, the vast majority of promoters were unmethylated in both normal colons and tumors (Fig. 4A), indicating open configurations^10^ and suggesting phenotypic plasticity. In contrast, enhancer methylation was bimodal, with lower mean methylation in tumors (46%) compared to normal colons (57%). Hence, the open chromatin at promoters in normal colon is largely maintained in tumors, and enhancers are less methylated or more open in tumors, potentially allowing for plasticity and rapid adaptation when cells encounter new microenvironments.

Finally, we sought to determine the relationship between DNA methylation and gene expression by performing RNA sequencing at the tumor gland scale. More precisely, mRNA levels were measured in four glands (two from each side) in 3 tumors (H, R, J). The mRNA expression correlation between glands on the same tumor side was larger compared to glands on opposite sides of the same tumor or glands from different tumors (Fig. 4B). In individual tumors, expression levels differed between glands, with 0.3-, 0.4- and 0.5- fold mean expression differences between glands in tumors J, H and R, respectively. However, there was no notable correlation between the degree of epigenetic conservation and RNA expression variability (Fig. 4C). In summary, although DNA methylation is conserved, gene expression is variable between glands, which may reflect phenotypic plasticity and adaptation of single growing tumor populations to heterogeneous local microenvironments.

## Discussion

How human tumors grow is difficult to ascertain because longitudinal observations are generally not feasible. Genome comparisons offer a practical way to reconstruct *in vivo* tumorigenesis because genomes record ancestries by becoming polymorphic. Multiregional sequencing studies reveal that mutational ITH is common in human tumors^1–3^, but it is uncertain if this genetic ITH is translated into stepwise selective sweeps during growth because most subclonal mutations are neutral passengers. In the case of human CRCs, the patterns of genetic ITH have been found to be more consistent with single neutral expansions, indicating that sequential stepwise selection may be uncommon during growth^6,8,11^. In this study, we performed multiregional epigenetic sampling and found that spatial epigenetic ITH is very limited, further corroborating that stepwise selection is infrequent or spatially limited to small sections during growth of human CRCs. Notably, DNA passenger methylation is also heterogeneous within individual tumor glands^25^ indicating that selective sweeps within glands are rare as well.

Although inferential, genome conservation is often used to identify functional regions under selection and is useful when direct observations or experimental manipulations are impractical. Indeed, epigenetic error rates during cell division are high compared to DNA error rates, and the resulting drift can create epigenetic ITH along the genome, with greater changes at non-functional CpG sites. As expected, we found preferential epigenetic conservation of highly expressed genes involved in core cellular functions, indicating that these genes are important in both normal colon and tumors. Interestingly, a search for loci that were specifically conserved in tumors but not in normal tissue revealed several genes that are involved in interferon signaling and antigen-processing and presentation.

Because HLA expression and tumor antigenicity are modulated by DNA methylation^26–28^, the observed conservation of IS genes stipulates a potential mechanism for evasion of immune surveillance during the growth of CRCs^29^. Whether immune surveillance is a significant extrinsic barrier to human tumorigenesis is unclear^19^. In the case of CRCs, T-cells reactive to neoantigens are present^30^ and mutations that could restrict neoantigen presentation to T-cells are rare^31^. Nevertheless, anti-PD-L1 therapies are ineffective against non-mutator phenotypes CRCs^32^, suggesting that other mechanisms such as chromatin regulation^33,34^ may contribute to evasion of immune surveillance. Finally, we note that our findings are consistent with recent CRISPR-Cas9 studies that found multiple IS genes influence immunoediting^35,36^.

The preferential conservation of IS gene methylation patterns between tumor sides – but not between tumor and normal tissues – implies that the founding tumor cells are under selection right from the start of tumor growth. Immune evasion may not be limited to tumors because mutations accumulate with age in normal colon^20^, and immune cytolytic activity is higher in the colon than in CRCs, with neoantigens depleted but not absent in CRCs^31^. Consistent with independent mutations and a customized mechanism where distinct neoantigens must be processed and presented differently to avoid immunoediting, IS gene methylation varied between different parts of normal adult colons. Because every founder cell lineage has a different set of neoantigens and must have previously evaded immunoediting, their progeny may simply perpetuate a successful incumbent strategy, resulting in the observed tumor-specific IS gene conservation.

The tumor microenvironment may play an important role in shaping the phenotypic ITH characterized by cells of different microscopic appearances and variable mRNA expression between regions or cells of the same CRCs^2,37,38^. If there was a strict correspondence between epigenome and phenotype, the observed epigenetic conservation would strongly limit phenotypic repertoire. However, phenotypic plasticity^21,22^ allows a single epigenome to confer multiple phenotypes, and broadly permissive chromatin in the colon allows crypt cells with different phenotypes to share similar epigenomes^23,24^. We found that the unmethylated promoters in normal colon were largely maintained in the tumors, and similar to other studies^39^, enhancers were more open in the tumors than adjacent normal tissue. Unmethylated promoters and more open enhancer configurations indicate that transcription factors induced by the microenvironment could potentially dictate tumor cell phenotypes. Hence, instead of waiting for stepwise evolution, a conserved but functionally plastic epigenome allows cells to immediately adapt to the many heterogeneous microenvironments encountered during growth. The ability to rapidly adapt rather than evolve through genetic bottlenecks may facilitate growth because tumor microenvironments may vary considerably temporally and spatially, even over a few millimeters^40^.

In conclusion, multiregional sampling reveals epigenetic ITH is minimal and more consistent with neutral evolution than frequent stepwise selection during primary human colorectal tumor growth. Importantly, this approach can characterize the degree of selection during growth, and epigenetic conservation can indicate which genes are more likely under selection. Our conclusions depend on highly purified tumor specimens because methylation differences can reflect variations in normal contaminating cells^41^. This epigenetic conservation provides new insights into the epigenome of the founder tumor cell whose stem like phenotypic plasticity can help explain the cellular heterogeneity and drug resistance of human tumors^21^. Unlike stepwise progression, where progenitors lack a complete set of drivers needed for robust growth, the founder cells of a neutral single expansion are already highly capable and adaptable, equipped with driver mutations and a largely open epigenome needed for rapid growth. Although further progression during growth may be rare, neutral evolution can be highly detrimental to the patient as it maximizes diversity^42^ and therefore increases the chances for further stepwise evolution (such as metastasis) and for selection of resistant subclones under therapeutic pressure. Taken together, both genetic^7,8^ and epigenetic multiregional measurements of human CRCs reconstruct portraits of founder cells that are born already poised for growth, phenotypic adaptation, and with an incumbent immune evasion strategy, helping to explain rapid expansions into visible “Big Bang” tumors.

## Methods

### Tumors

The tumor (n = 16) and matched normal colon (n = 6) samples (Table 1) were obtained fresh at the Norris Comprehensive Cancer Center, and samples (~0.5 cm^3^) from opposite tumor sides and from different parts of the normal colons were procured, as previously described^7^. The specimens were procured in accordance with relevant guidelines and regulations, and all experimental protocols were approved by the Institutional Review Board at the University of Southern California. Informed consent was not obtained because the specimens were considered exempt by our Institutional Review Board. Other data from 10 of the tumors have been previously published^7^ and have the same names (except the tumor originally labeled *R*). From each bulk sample (tumor or normal colon), individual tumor glands (10,000 to 30,000 cells) or intestinal crypts were isolated by an EDTA washout that results in >90% epithelial cell purity^43^. Examples of isolated tumor glands from our study and corresponding LUMP purity estimates are provided in Fig. S1. Bulk methylation levels were measured by pooling hundreds of individual tumor glands or normal colon crypts.

### DNA methylation

Bulk sample DNA methylation was measured using the EPIC methylation array (Illumina) for opposite sides of 16 tumors, gland pairs from opposite sides of 11 CRCs, and six normal colons. Idat files in each color channel (red and green) were processed with the ‘noob’ function in the minfi R program to extract the methylated and unmethylated probe signal intensities and to calculate the beta value for each data point after within-array normalization for background correction, dye-bias equalization and probe design. Beta values between samples were compared based on pairwise distances (PWDs) defined as the absolute differences in beta values. Only autosomal CpG sites were analyzed.

Methylation of four widely spaced samples from nine normal adult colons (ages 50 to 70 years, mucosal biopsies, not EDTA washout-purified) was also measured with the Infinium methylation arrays (Illumina). Raw intensity data was read, preprocessed and batch corrected using the minfi (v.1.16.0) in R (v3.2.2). Probes were mapped to the genome and probes showing mean intensity p-value > 0.05 across all samples were excluded. Excluded probes also included: probes with proximal SNPs; non-CpG probes; probes on sex chromosomes; probes with observed cross-reactivity between two or more genomic regions. After subsequent filtering, 426,718 probes were left for analysis.

### Gene annotation

The CpG sites were classified based on the UCSC annotations provided with the array. Enhancers were defined by FANTOM5^44^. The panel of genes with higher expression in CRCs (RPKM > 1) was obtained from the non-hypermutated data in Supplemental Table 3 in ref.^9^.

### Pathway enrichment analysis

Autosomal genes with at least four annotated CpG sites (N = 22,430) were classified based on methylation differences between normal colon and tumor (average colon methylation versus average tumor methylation) and between sides (PWD between tumor sides). There were 5,988 genes consistently (90% or more of tumors) with the four types of conservation in Fig. 3A and Table 2. The gene lists were analyzed with the Reactome pathway enrichment algorithm^18^. A false detection rate (FDR) cutoff of <0.05 was used.

Another method to find genes preferentially conserved only during tumor growth identified genes with average PWDs greater than 0.075 between normal colons from different patients, between normal and tumor from the same patient, and between tumors from different patients, but with PWDs less than 0.05 between opposite tumor sides from the same patient. The gene lists from average and individual tumor data were analyzed with the Reactome algorithm.

### RNA measurements

RNA was measured using the NanoString GX Human Cancer Reference Kit which measures 230 cancer-related RNAs. Single tumor glands (~10,000 cells each, 2 glands from each side of tumors H, J, R from Table 1) were analyzed directly. Data were normalized for each tumor using the geometric mean with nSolver 3.0 software. To minimize measurement variability, only genes with at least 30 counts in all four glands and with CpG sites in the EPIC array (136 genes for J, 101 genes for R, 84 gene for H) were compared.

### Cell line studies

A single cell was isolated from a bulk tissue culture of HCT116 (American Type Culture Collection), grown to confluence, and split into three flasks. Each of the three parallel lines were serially cultured for 18 to 24 passages (52 to 94 days), 70 passages (409 days) and 105 passages (682 days). DNA methylation was measured using EPIC arrays and compared between the samples.

### Xenograft studies

Single cells were isolated from bulk tissue cultures and grown to several million cells. About 1 million cells were injected subcutaneously into immune deficient mice (nu/nu) and xenografts (~1 cm^3^) were harvested within 3 months after injection. DNA was isolated from physically distinct clonal xenografts (separate flanks or different mice) derived from HCT116 (2 xenografts), SW480 (two xenografts) and LoVo (three xenografts). These mouse experiments were performed in accordance with relevant guidelines and regulations, and were approved by the Institutional Animal Care and Use Committee at the University of Southern California. DNA methylation was measured using EPIC arrays.

## Electronic supplementary material

Supplemental Information

## References

[1] Gerlinger M (2012). Intratumor heterogeneity and branched evolution revealed by multiregion sequencing. N. Engl. J. Med..

[2] McGranahan Nicholas, Swanton Charles (2017). Clonal Heterogeneity and Tumor Evolution: Past, Present, and the Future. Cell.

[3] Shibata D (2012). Cancer. Heterogeneity and tumor history. Science.

[4] Andor N (2016). Pan-cancer analysis of the extent and consequences of intratumor heterogeneity. Nat. Med..

[5] Vogelstein B (2013). Cancer genome landscapes. Science.

[6] Ryser MD, Min BH, Siegmund KD, Shibata D (2018). Spatial mutation patterns as markers of early colorectal tumor cell mobility. Proc. Natl. Acad. Sci. USA.

[7] Sottoriva A (2015). A Big Bang model of human colorectal tumor growth. Nat. Genet..

[8] Sun R (2017). Between-region genetic divergence reflects the mode and tempo of tumor evolution. Nat. Genet..

[9] Williams MJ, Werner B, Barnes CP, Graham TA, Sottoriva A (2016). Identification of neutral tumor evolution across cancer types. Nat. Genet..

[10] Jones PA (2012). Functions of DNA methylation: islands, start sites, gene bodies and beyond. Nat. Rev. Genet..

[11] Shlyueva D, Stampfel G, Stark A (2014). Transcriptional enhancers: from properties to genome-wide predictions. Nat. Rev. Genet..

[12] Yang X (2014). Gene body methylation can alter gene expression and is a therapeutic target in cancer. Cancer Cell..

[13] Cancer Genome Atlas Network. Comprehensive molecular characterization of human colon and rectal cancer. *Nature* **487**, 330–337 (2012). 10.1038/nature11252 PMC3401966 22810696

[14] Martínez-Cardús A (2016). Epigenetic homogeneity within colorectal tumors predicts shorter relapse-free and overall survival times for patients with locoregional cancer. Gastroenterology.

[15] Uchi R (2016). Integrated multiregional analysis proposing a new model of colorectal cancer evolution. PLoS Genet..

[16] Roerink SF (2018). Intra-tumour diversification in colorectal cancer at the single-cell level. Nature.

[17] Shibata D, Tavare S (2006). Counting divisions in a human somatic cell tree: how, what and why?. Cell Cycle.

[18] Fabregat A (2016). The Reactome pathway Knowledgebase. Nucleic Acids Res..

[19] Schumacher TN, Schreiber RD (2015). Neoantigens in cancer immunotherapy. Science.

[20] Blokzijl F (2016). Tissue-specific mutation accumulation in human adult stem cells during life. Nature.

[21] Easwaran H, Tsai HC, Baylin SB (2014). Cancer epigenetics: tumor heterogeneity, plasticity of stem-like states, and drug resistance. Mol. Cell.

[22] Meacham CE, Morrison SJ (2013). Tumour heterogeneity and cancer cell plasticity. Nature.

[23] Kaaij LT (2013). DNA methylation dynamics during intestinal stem cell differentiation reveals enhancers driving gene expression in the villus. Genome Biol..

[24] Kim TH (2014). Broadly permissive intestinal chromatin underlies lateral inhibition and cell plasticity. Nature.

[25] Siegmund KD, Marjoram P, Tavare S, Shibata D (2011). High DNA methylation pattern intratumoral diversity implies weak selection in many human colorectal cancers. PLoS One.

[26] Campoli M, Ferrone S (2008). HLA antigen changes in malignant cells: epigenetic mechanisms and biologic significance. Oncogene.

[27] Fonsatti E (2007). Functional up-regulation of human leukocyte antigen class I antigens expression by 5-aza-2′-deoxycytidine in cutaneous melanoma: immunotherapeutic implications. Clin. Cancer Res..

[28] Siebenkas C (2017). Inhibiting DNA methylation activates cancer testis antigens and expression of the antigen processing and presentation machinery in colon and ovarian cancer cells. PLoS One.

[29] DuPage M, Mazumdar C, Schmidt LM, Cheung AF, Jacks T (2012). Expression of tumour-specific antigens underlies cancer immunoediting. Nature.

[30] Tran E (2015). Immunogenicity of somatic mutations in human gastrointestinal cancers. Science.

[31] Rooney MS, Shukla SA, Wu CJ, Getz G, Hacohen N (2015). Molecular and genetic properties of tumors associated with local immune cytolytic activity. Cell.

[32] Le DT (2015). PD-1 Blockade in Tumors with Mismatch-Repair Deficiency. N. Engl. J. Med..

[33] Miao D (2018). Genomic correlates of response to immune checkpoint therapies in clear cell renal cell carcinoma. Science.

[34] Pan D (2018). A major chromatin regulator determines resistance of tumor cells to T cell-mediated killing. Science.

[35] Manguso RT (2017). *In vivo* CRISPR screening identifies Ptpn2 as a cancer immunotherapy target. Nature.

[36] Patel SJ (2017). Identification of essential genes for cancer immunotherapy. Nature.

[37] Dunne PD (2017). Cancer-cell intrinsic gene expression signatures overcome intratumoural heterogeneity bias in colorectal cancer patient classification. Nat. Commun..

[38] Li H (2017). Reference component analysis of single-cell transcriptomes elucidates cellular heterogeneity in human colorectal tumors. Nat. Genet..

[39] Akhtar-Zaidi B (2012). Epigenomic enhancer profiling defines a signature of colon cancer. Science.

[40] Heindl A, Nawaz S, Yuan Y (2015). Mapping spatial heterogeneity in the tumor microenvironment: a new era for digital pathology. Lab. Invest..

[41] Aran D, Sirota M, Butte AJ (2015). Systematic pan-cancer analysis of tumour purity. Nat. Commun..

[42] Ling S (2015). Extremely high genetic diversity in a single tumor points to prevalence of non-Darwinian cell evolution. Proc. Natl. Acad. Sci. USA.

[43] Nakamura S, Goto J, Kitayama M, Kino I (1994). Application of the crypt-isolation technique to flow-cytometric analysis of DNA content in colorectal neoplasms. Gastroenterology.

[44] Andersson R (2014). An atlas of active enhancers across human cell types and tissues. Nature.

