# Supplementary material for: Epigenetic Heterogeneity in Human Colorectal Tumors Reveals Preferential Conservation And Evidence of Immune Surveillance
Source: Sci Rep. 2018 Nov 23;8:17292. doi: 10.1038/s41598-018-35621-y (PMC6251922; doi:10.1038/s41598-018-35621-y)
Supplement: Supplementary file 1 — Supplemental Information [file 41598_2018_35621_MOESM1_ESM.pdf]

Supplemental File For:

Epigenetic Heterogeneity in Human Colorectal Tumors Reveals Preferential Conservation And Evidence of Immune Surveillance

Marc D. Ryser, Ming Yu, William Grady, Kimberly Siegmund, Darryl Shibata

Tumor	Reactome enriched (FDR < 0.05)
**K**	B2M;HLA-A;HLA-C;HLA-F;HLA-H;IRF7;ISG20;OAS1
**S**	B2M;CYBA;HLA-A;HLA-B;HLA-C;HLA-F;HLA-H;IRS1;NAT1;PSMD5;ASB6;DTX3L;TRIM4;CD74; FOXO1;HIST1H3F;HIST1H3G;HIST1H3J;HIST3H3;PPP2R1B;RAC3
**P**	B2M;CYBA;FLNB;HLA-A;HLA-B;HLA-C;HLA-H;IRF7;ISG20;ITGB5;KPNA1;OAS1;PSMD5;SOCS3; TRIM2;TRIM6
**X**	B2M;CYBA;FLNB;GBP4;HLA-A;HLA-B;HLA-C;HLA-F;HLA-H;IRF7;ISG20;ITGB5;KPNA1;NAT1; OAS1;PSMD5;SOCS3;TRIM2;TRIM6
**O**	B2M;CYBA;FLNB;HLA-A;HLA-B;HLA-C;HLA-F;HLA-H;ISG20;ITGB5;KPNA1;NAT1;OAS1;PSMD5; TRIM6
**C**	B2M;FLNB;GBP4;HLA-A;HLA-B;HLA-H;IRF7;ISG20;ITGB5;NAT1;OAS1;PSMD5;SOCS3;TRIM2
**H**	B2M;CYBA;HLA-A;HLA-B;HLA-F;HLA-H;PSMD5;ASB6;BTBD6;DTX3L;SMURF1;TRIM4;UBR4
**J**	GBP4;HLA-A;HLA-F;HLA-H;IRF7;TRIM6
**M**	B2M;CYBA;FLNB;HLA-A;HLA-B;HLA-C;HLA-F;HLA-H;IRF7;ISG20;ITGB5;KPNA1;NAT1;OAS1; PSMD5;SOCS3;TRIM2;TRIM6
**T**	B2M;FLNB;GBP4;HLA-B;HLA-C;HLA-F;HLA-H;ISG20;ITGB5;KPNA1;NAT1;OAS1;PSMD5;SOCS3; TRIM2;TRIM6
**R**	B2M;CYBA;FLNB;GBP4;HLA-A;HLA-B;HLA-C;HLA-F;HLA-H;IRF7;ISG20;ITGB5;KPNA1;NAT1; OAS1;PSMD5;TRIM2;TRIM6
**F**	B2M;CYBA;FLNB;GBP4;HLA-A;HLA-B;HLA-C;HLA-F;HLA-H;IRF7;ISG20;ITGB5;KPNA1;OAS1; PSMD5;SOCS3;TRIM2;TRIM6;BTBD6;DTX3L;SMURF1;TRIM4;UBR4;AMICA1;ATG7;CUL3; CXADR;FBXL22;HACE1;KLHL2;KLHL3;LMO7;LNX1; NEDD4L; SH3RF1;SPSB1;ZNRF1;ZNRF2
**W**	B2M;CYBA;FLNB;GBP4;HLA-A;HLA-C;HLA-H;NAT1;PSMD5;SOCS3
**D**	B2M;CYBA;GBP4;HLA-A;HLA-B;HLA-C;HLA-F;HLA-H;KPNA1;OAS1;PSMD5;SOCS3;BTBD6; DTX3L;TRIM4;FOXO1;HIST1H3F;HIST1H3G;HIST3H3;PPP2R1B;FBXL22;LNX1;NEDD4L;SPSB1; ZNRF2;ARTN;CASP1;CUL3;DUSP2;EGFR;FAN1;FGF18;GRIN2D;HBEGF;HIST2H3D;HMOX1; IL18; IRS2;LTBR;LYN;MAP2K6;MIR6852;MUC1;PIK3R1;PPP2CB;STAT5B;TNFRSF12A; TNFRSF1B;TNFSF9
**U**	B2M;CYBA;FLNB;GBP4;HLA-A;HLA-B;HLA-C;HLA-F;IRF7;ISG20;ITGB5;KPNA1;OAS1;PSMD5; SOCS3;TRIM2;TRIM6;BTBD6;DTX3L;SMURF1;TRIM4;UBR4;AMICA1;ATG7;CUL3;CXADR; FBXL22; HACE1;KLHL2;KLHL3;LMO7;LNX1;NEDD4L;SH3RF1;SPSB1;ZNRF1
**E**	none in antigen processing pathways
**Aggregate***	B2M;CYBA;FLNB;GBP4;HLA-A;HLA-B;HLA-C;HLA-F;HLA-H;IRF7;ISG20;ITGB5;KPNA1;NAT1; OAS1;PSMD5;SOCS3;TRIM2

Table S1: Reactome enriched genes in immune presentation pathways *Reactome run with pairwise distance averages of all 16 tumors

Figure S1. A) Bright-field examples of intact tumor glands isolated from fresh bulk tumor tissues with an EDTA-washout method. The glands are largely free of stromal cells, with tumor cell purity estimated at more than 90%. **B)** We used the LUMP algorithm (Aran D, Sirota M, Butte AJ. Nat Commun. 2015;6:8971) to estimate tumor purity. This method is based on 44 CpG sites that tend to be unmethylated (<5%) in blood cells and methylated (>30%) in cancer cells. 42 of the 44 CpG sites are shared with the EPIC 850K array. Using these 42 sites, the human bulk specimens had an average tumor purity of 87% (inter-quartile range [IQR]: 80%-100%, range: 62%-100%), further documenting the relatively high percentages of tumor cells that can be obtained with the EDTA-washout method.

Figure S2. Violin plots showing the corresponding full distributions of the pairwise distance estimates between different sample types as shown in Figure 2F. Note that in Fig 2F, the means of the full distributions are shown only.

A

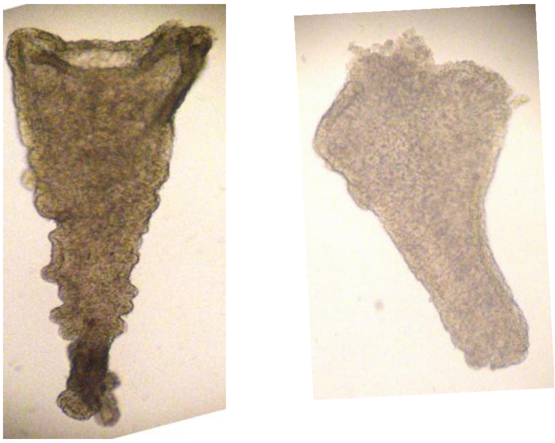

B

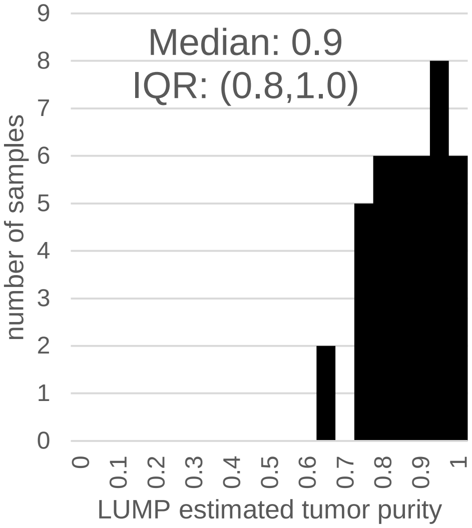
Median: 0.9
IQR: (0.8,1.0)
number of samples
0
1
2
3
4
5
6
7
8
9
0
0.1
0.2
0.3
0.4
0.5
0.6
0.7
0.8
0.9
1
LUMP estimated tumor purity

Figure S1

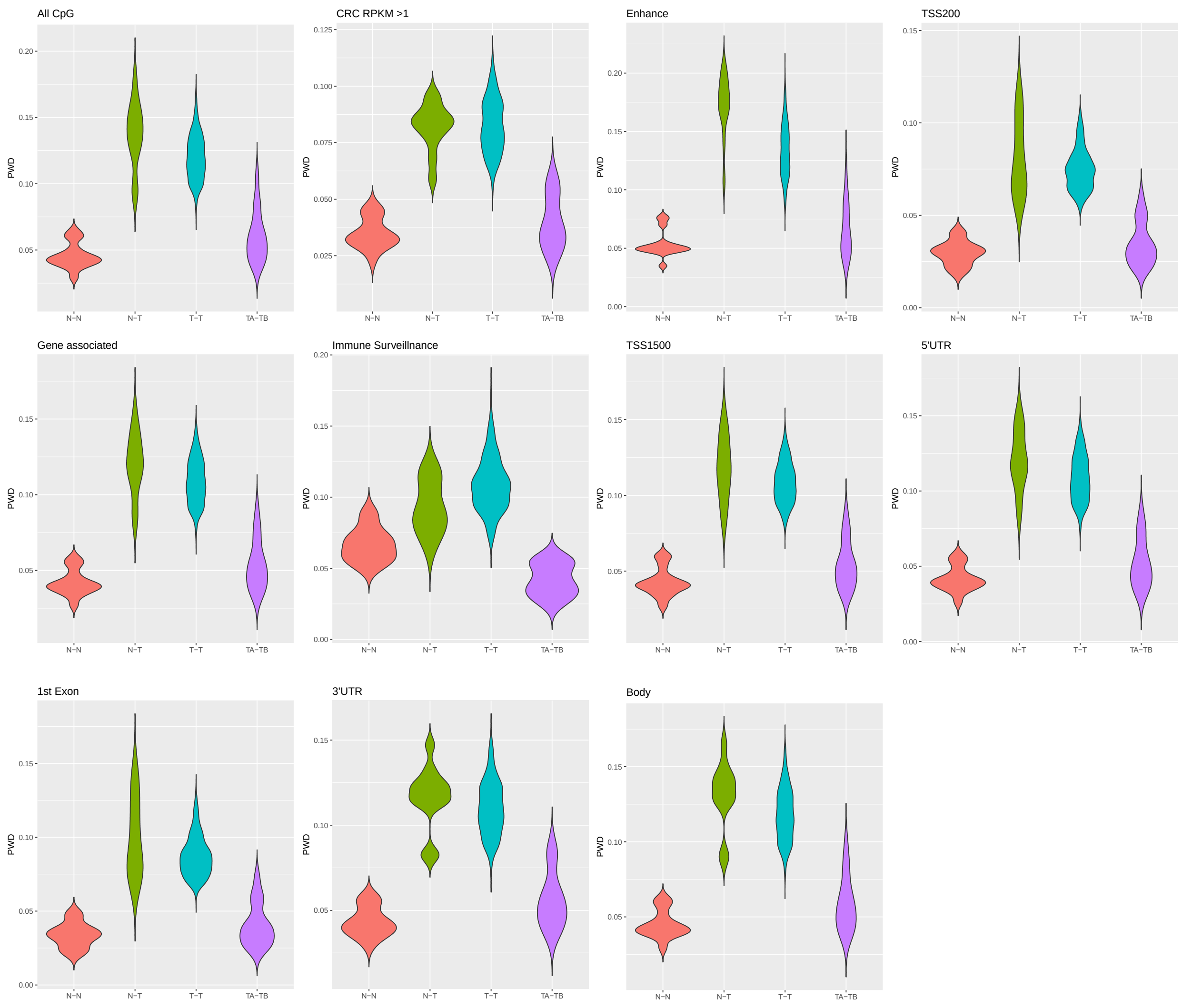
All CpG
CRC RPKM >1
Enhance
TSS200
Gene associated
Immune Surveillnance
TSS1500
5'UTR
1st Exon
3'UTR
Body
PWD
N−N
N−T
T−T
TA−TB

Figure S2
